# Association of Prehospital Epinephrine Administration With Survival Among Patients With Traumatic Cardiac Arrest Caused By Traffic Collisions

**DOI:** 10.1038/s41598-019-46460-w

**Published:** 2019-07-09

**Authors:** Makoto Aoki, Toshikazu Abe, Kiyohiro Oshima

**Affiliations:** 1 Department of Emergency Medicine, Gunma University Graduate School of Medicine, Gunma, Japan; 2 Department of General Medicine, Juntendo University, Tokyo, Japan; 3 Department of Health Services Research, Faculty of Medicine, University of Tsukuba, Ibaraki, Japan

**Keywords:** Epidemiology, Outcomes research

## Abstract

For traumatic cardiac arrest (TCA), the effect of prehospital epinephrine administration was unclear. The aim of this study was to evaluate the relationship between prehospital epinephrine administration and survival in patients with TCA caused by traffic collisions. We conducted a nationwide, prospective, population-based observational study involving patients who experienced out-of-hospital cardiac arrest (OHCA) by using the All-Japan Utstein Registry. Blunt trauma patients with TCA who received prehospital epinephrine were compared with those who did not receive prehospital epinephrine. The primary outcome was 1-month survival of patients. The secondary outcome was prehospital return of spontaneous circulation (ROSC). A total of 5,204 patients with TCA were analyzed. Of those, 758 patients (14.6%) received prehospital epinephrine (Epinephrine group), whereas the remaining 4,446 patients (85.4%) did not receive prehospital epinephrine (No epinephrine group). Eleven (1.5%) and 41 (0.9%) patients in the Epinephrine and No epinephrine groups, respectively, survived for 1 month. In addition, 74 (9.8%) and 40 (0.9%) patients achieved prehospital ROSC in the Epinephrine and No epinephrine groups, respectively. In multivariable logistic regression models, prehospital epinephrine administration was not associated with 1-month survival (odds ratio [OR] 1.495, 95% confidence interval [CI] 0.758 to 2.946) and was associated with prehospital ROSC (OR 3.784, 95% CI 2.102 to 6.812). A propensity score-matched analysis showed similar results for 1-month survival (OR 2.363, 95% CI 0.606 to 9,223) and prehospital ROSC (OR 6.870, 95% CI 3.326 to 14.192). Prehospital epinephrine administration in patients with TCA was not associated with 1-month survival, but was beneficial in regard to prehospital ROSC.

## Introduction

Various treatments have been developed to decrease the mortality caused by trauma^1^. However, the mortality rate related to traumatic cardiac arrest (TCA) remains high and the latest systematic review reported the mortality was 97.6%^2–9^. Prehospital advanced life support (ALS) is currently widely used. An observational study reported that prehospital ALS was associated with survival of patients with TCA^10^. However, the component of prehospital ALS associated with survival is unknown^11^.

Recently, numerous observational studies and the latest randomized control trial of endogenic cardiac arrest reported that prehospital epinephrine administration was associated with patient survival^12–14^. However, few TCA patients were included in this randomized control trial.

Resuscitation algorithm for TCA was naturally introduced from the immediate treatment of reversible causes such as resuscitative thoracotomy and did not provide obvious opinion of epinephrine administration in patients with TCA^15–17^. There is limited evidence regarding the effectiveness of prehospital epinephrine administration in patients with TCA, except one small observational study^11^. The study revealed that prehospital administration of epinephrine in patients with TCA was associated with increased survival-to-discharge. However, half of the patients who survived in this study were penetrating trauma patients. Thus, the efficacy of prehospital epinephrine administration in blunt trauma patients with TCA was unknown.

The objective of the present study was to investigate the efficacy of prehospital epinephrine administration in blunt trauma patients with TCA.

## Methods

### Study design and setting

This study was a post-hoc analysis of data from the All-Japan Utstein Registry – a prospective, nationwide, population-based out-of-hospital cardiac arrest (OHCA) registry system based on the Utstein style – established by the Fire and Disaster Management Agency^18,19^. This OHCA registry includes all patients with TCA caused by traffic collisions, who were treated by the emergency medical service (EMS) from January 1, 2012 to December 31, 2015. The analysis protocol of this study was approved by the Ethics Committee of the Gunma University Hospital.

Cardiac arrest was defined as the cessation of cardiac mechanical activity, confirmed by the absence of signs of circulation^18,19^. The cause of arrest was presumed to be of traumatic origin based on circumstantial evidence. Confirmation of the traumatic origin of cardiac arrest was clinically determined by the treating physicians who collaborated with the EMS personnel.

### EMS system in japan and data collection

The free emergency telephone number 1-1-9 is used to call an ambulance from anywhere in Japan. Emergency services are provided 24 hours per day. Following a call, an ambulance is dispatched from the nearest fire station. In general, an ambulance includes a crew of three EMS personnel. Most ambulances include at least one emergency life-saving technician (ELST), certified to insert an intravenous catheter and an adjunct airway and use semiautomated external defibrillators in patients with OHCA. In addition, since July 2004 and April 2006, specially trained ELST are certified to insert an endotracheal tube and administer intravenous epinephrine. In 2014, almost all (97.4%) ambulances included at least one ELST and the vast majority of those (82.9%) were specially trained ELSTs^20^. However, these ALS procedures cannot be performed without the instruction of a medical director in each municipality. The treatment of cardiac arrest is based on the Japanese cardiopulmonary resuscitation (CPR) guidelines, derived from those established by the American Heart Association, the European Resuscitation Council, and the International Liaison Committee on Resuscitation guidelines. EMS providers are not permitted to terminate resuscitation in the field^21^. Therefore, most patients with OHCA are treated by EMS personnel, transported to a hospital, and registered in the All-Japan Utstein Registry.

### Patient selection

**Figure 1:**
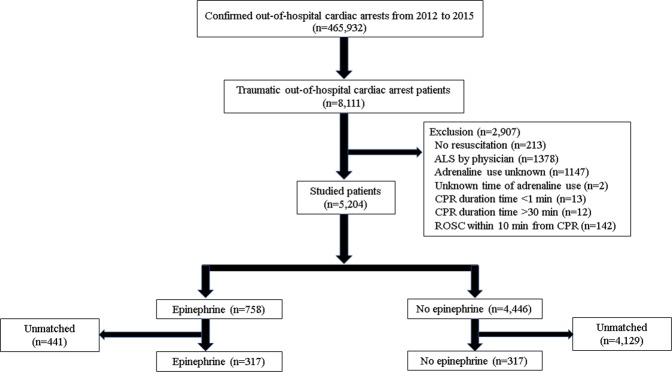
Flow chart of patients included in this study. ALS, advanced life support; CPR, cardiopulmonary resuscitation; ROSC, return of spontaneous circulation.

The patient flow chart is shown in Fig. 1. Exclusion criteria were: no resuscitation, ALS performed by a physician (i.e., bias from another ALS procedure such as blood transfusion, insertion of chest tube, thoracotomy, resuscitative endovascular occlusion of the aorta, etc.), unknown adrenaline use, CPR duration time <1 min, CPR duration time >30 min (i.e., unsurvivable long time), and prehospital return of spontaneous circulation (ROSC) within 10 min from CPR (i.e., bias caused by inclusion of patients successfully resuscitated prior to epinephrine administration).

### Data collection

Data regarding patient age, gender, type of bystander witness status, first recorded cardiac rhythm, life support by EMS personnel (i.e., use of ALS devices, insertion of an intravenous line), time course of resuscitation and epinephrine administration, prehospital ROSC, and 1-month survival were obtained. Data regarding EMS times of call receipt, arrival of the ambulance at the scene of the accident, contact with patients, initiation of CPR, defibrillation performed by EMS personnel, and arrival at the hospital were recorded using the clock of each EMS system. In cases of shock delivery by bystanders using a public-access automated external defibrillator (AED), the patient’s first recorded rhythm was regarded as ventricular fibrillation or pulseless ventricular tachycardia. The estimated times of collapse, initiation of public-access AED shocks, and initiation of bystander CPR were obtained through an interview with the bystander performed by the EMS personnel. The type of bystander CPR was obtained through observation and interview with the bystander performed by the EMS personnel before leaving the scene of the accident. A set of specific questions on the presence or absence of chest compressions and rescue breathing was used for this purpose. The time interval from collapse to shocks using public-access AEDs was replaced with time to bystander CPR, unless the time of shocks delivered using public access AEDs was available. Patients who survived OHCA were followed up for a maximum of 1 month after the event by the EMS personnel in charge. The data forms were completed by the EMS personnel in cooperation with the treating physicians. The data were integrated into the All-Japan Utstein Registry database server and logically checked by the computer system. In cases of incomplete data forms, the Fire and Disaster Management Agency requested the provision of missing data from the respective fire station.

### Outcomes

The primary outcome was 1-month survival. The secondary outcome was prehospital return of spontaneous circulation (ROSC).

### Statistical analysis

Continuous variables were expressed as medians (interquartile range). The patients were divided into two groups, namely Epinephrine and No epinephrine. Comparisons of continuous variables between the Epinephrine and No epinephrine groups were performed using the Mann‒Whitney U test. Categorical variables were expressed as counts and percentages, and comparisons of each categorical variable between groups were performed using the chi-squared test. Outcomes were evaluated using univariate and multiple logistic regression analyses to assess the independent effect of epinephrine administration. Confounders for multiple logistic regression models were selected on the basis of the assumption that these were directly affected by epinephrine administration (according to previous reports) and their clinical importance^11^. Covariates of the primary outcome included age, gender, first rhythm and time from the call to arrival at the scene of the accident. The presence of a witness was not included in the covariates because this was evaluated in the subgroup analysis. We set two multiple logistic regression models of the secondary outcome. In the first model we selected the covariates of the primary analysis, while in the second model we included age, gender, first rhythm, use of ALS devices, insertion of an intravenous line, and time from the call to arrival at the scene of the accident to adjust the differences between two groups more precisely. In addition, multicollinearity was assessed using a variance inflation factor and the tolerance value was set at <2. Moreover, the goodness-of-fit was assessed using the Hosmer–Lemeshow test. Statistical significance was defined as a two-sided *P* < 0.05 or assessed using a 95% confidence interval (CI) in all statistical analyses. All statistical analyses, excluding the propensity score (PS) matching were performed using the IBM SPSS Statistics version 23.0 (SPSS Inc, Chicago, Illinois, USA).

### Propensity score

We chose the PS-matching analysis because prehospital epinephrine administration was not randomly assigned. A logistic regression analysis was performed to estimate a PS for the prediction of prehospital epinephrine administration from the available predictors. Confounders for the PS were selected according to a previous report^11^. Clinically important confounders were included in the calculation of the PS. These variables were age, gender, presence of a witness, bystander CPR, first rhythm, use of ALS devices, insertion of an intravenous line, and time from the call to arrival at the scene of the accident. PS matching extracted 1:1 matched pairs of patients using a caliper with 0.0005 with administration/or not of epinephrine based on the averaged PS. The absolute standardized difference of variables for the estimation of PS was used to assess the match balance, whereby an absolute standardized difference >0.1 represented a meaningful imbalance. In the PS-matched cohort, a univariate logistic regression analysis was performed to evaluate the association between epinephrine administration and outcomes. PS matching was performed using the R software for Windows (version 3.4.0; R Foundation for Statistical Computing, Vienna, Austria).

### Subgroup analysis

Subgroup analysis was performed to identify the potential benefits and demerits of prehospital epinephrine administration. Patients with TCA and presence of a witness were selected. In the subgroup analysis, we performed PS matching. The variables used to calculate the PS were age, gender, bystander CPR, first rhythm, use of ALS devices, insertion of an intravenous line, and time from the call to arrival at the scene of the accident. In the PS-matched cohort, a univariate logistic regression analysis was performed.

### Sensitivity analysis

We performed a sensitivity analysis involving patients with TCA who achieved prehospital ROSC within 10 min from CPR to verify that the results were not altered by the selection of patients. In the sensitivity analysis, we performed PS matching. The variables used to calculate the PS were age, gender, bystander CPR, first rhythm, use of ALS devices, insertion of an intravenous line, and time from the call to arrival at the scene of the accident. In the PS-matched cohort, a univariate logistic regression analysis was performed.

### Ethics approval and consent to participate

The analysis protocol of this study was approved by the Ethics Committee of the Gunma University Hospital. Because of the anonymous and retrospective nature of the study, the need for informed consent was waived.

## Results

A total of 465,932 patients with OHCA were documented from January 1, 2012 to December 31, 2015. Among those, cardiac arrest was presumed to be of traumatic origin caused by traffic collision in 8,111 patients. We identified a total of 5,204 patients with TCA eligible for analysis (Fig. 1). Of those, 758 patients received prehospital epinephrine (Epinephrine group), whereas the remaining 4,446 patients did not receive prehospital epinephrine (No epinephrine group). Patient characteristics are shown in Table 1. The median age of patients was 61 years (40-75 years). Age was significantly higher in the Epinephrine group versus the No epinephrine group (64 [44–77] years vs. 61 [39–75] years, respectively; *P* = 0.004). The majority of patients were male (69%; 3,599/5,204 patients). The percentages of witness presence and bystander CPR were significantly higher in the Epinephrine group versus the No epinephrine group (witness: 567/758 patients [75%] vs. 3,053/4,446 patients [69%], respectively; *P* = 0.001 and bystander CPR: 195/758 patients (26%) vs. 796/4,446 patients (18%), respectively; *P* < 0.001). Approximately 92% of the patients exhibited a non-shockable first rhythm. There were significantly more non-shockable first rhythms in Epinephrine group than No epinephrine group. Regarding life support provided by EMS personnel, use of ALS devices and insertion of an intravenous line were more frequent in the Epinephrine group (use of ALS devices: 469/758 patients (62%) vs. 1,049/4,446 patients (24%), respectively; *P* < 0.001 and insertion of an intravenous line: 739/758 patients (98%) vs. 480/4,446 patients (11%), respectively; *P* < 0.001). Both times from the call to arrival at the scene of the accident and at the hospital were significantly longer in the Epinephrine group (call to arrival at the scene of the accident: 8 (6–11) min vs. 8 (6–10) min, respectively; *P* < 0.001 and call to arrival at the hospital: 38 (30–48) min vs. 32 (25–43) min, respectively; *P* < 0.001).

**Table 1:** Baseline characteristics of the patients with traumatic cardiac arrest according to epinephrine administration (n = 5,204).

Characteristics	Epinephrine (n = 758)	No epinephrine (n = 4,446)	*P* value
Age, median (IQR), y	64 (44–77)	61 (39–75)	0.004
≦17	19 (2.5%)	211 (4.7%)	0.006
18–64	370 (49%)	2258 (51%)	
≧65	369 (49%)	1977 (45%)	
Gender			0.277
Male	537 (71%)	3062 (69%)	
Female	221 (29%)	1384 (31%)	
Witness			0.001
Unwitnessed	191 (25%)	1393 (31%)	
Witnessed	567 (75%)	3053 (69%)	
Bystander CPR			<0.001
Any CPR	195 (26%)	796 (18%)	
No CPR	563 (74%)	3650 (82%)	
First rhythm			<0.001
Ventricular fibrillation	21 (2.8%)	82 (1.8%)	
Pulseless ventricular tachycardia	2 (0.3%)	9 (0.2%)	
Pulseless electrical activity	341 (45%)	1482 (33%)	
Asystole	354 (47%)	2618 (59%)	
Other	40 (5.3%)	255 (5.7%)	
**Life support by emergency medical personnel**			
Use of advanced life support devices	469 (62%)	1049 (24%)	<0.001
Insertion of intravenous line	739 (98%)	480 (11%)	<0.001
Time from call to arrival at scene	8 (6–11)	8 (6–10)	<0.001
Time from call to arrival at hospital	38 (30–48)	32 (25–43)	<0.001

IQR; interquartile range, CPR; cardiopulmonary resuscitation.

Missing; Time from call to arrival at scene = 13 and Time from call to arrival at hospital = 13.

**Figure 2:**
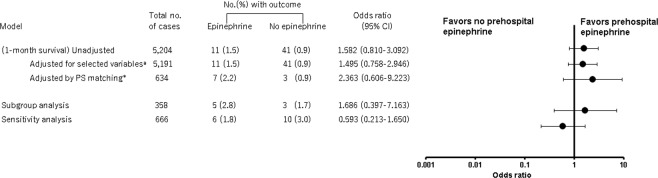
Effect of prehospital epinephrine administration on 1-month survival. (**a**) The adjusted confounders included age, gender, first rhythm, and time from the call to arrival at the scene of the accident. (**b**) The covariates used to estimate the PS were age, gender, witness, bystander CPR, first rhythm, advanced life support devices, insertion of an intravenous line, and time from call to arrival at the scene of the accident. CI, confidence interval; CPR, cardiopulmonary resuscitation; PS, propensity score.

**Figure 3:**
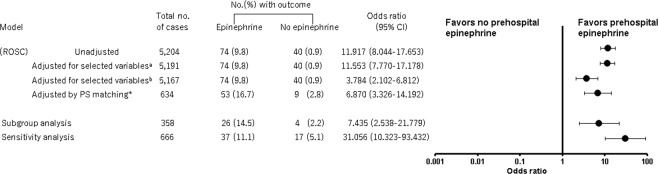
Effect of prehospital epinephrine administration on return of spontaneous circulation. (**a**) The adjusted confounders included age, gender, first rhythm, and time from the call to arrival at the scene of the accident. (**b**) The adjusted confounders included age, gender, first rhythm, advanced life support devices, insertion of an intravenous line, and time from the call to arrival at the scene of the accident. (**c**) The covariates used to estimate the PS were age, gender, witness, bystander CPR, first rhythm, advanced life support devices, insertion of an intravenous line, and time from the call to arrival at the scene of the accident. CI, confidence interval; ROSC, return of spontaneous circulation; CPR, cardiopulmonary resuscitation; PS, propensity score.

Figures 2 and 3 show the comparison of outcomes between the Epinephrine and the No epinephrine groups. There was no significant difference in 1-month survival between the groups (1.5% [11/758 patients] vs. 0.9% [41/4,446 patients], respectively; *P* = 0.176). However, the rate of prehospital ROSC was higher in the Epinephrine group (9.8% [74/758 patients] vs. 0.9% [40/4,446 patients], respectively; *P* < 0.001). The multiple logistic regression analysis showed that prehospital epinephrine administration was not associated with 1-month survival (odds ratio [OR] 1.495, 95% CI 0.758 to 2.946); however, it was associated with prehospital ROSC (OR 3.784, 95% CI 2.102 to 6.812) (Figs 2 and 3).

Table 2 shows the baseline characteristics of PS-matched patients. The characteristics of PS-matched patients were balanced in terms of absolute standardized mean difference (<0.1 between the groups). In PS-matched patients, there was no significant difference in 1-month survival between the Epinephrine and No epinephrine groups (2.2% [7/317 patients] vs. 0.9% [3/317 patients], respectively; *P* = 0.340). However, the rate of prehospital ROSC was higher in the Epinephrine group (16.7% [53/317 patients] vs. 2.8% [9/317 patients], respectively; *P* < 0.001). Prehospital epinephrine administration was not associated with 1-month survival (OR 2.363, 95% CI 0.606 to 9,223); however, it was associated with prehospital ROSC (OR 6.870, 95% CI 3.326 to 14.192) (Figs 2 and 3).

**Table 2:** Baseline characteristics of the propensity score-matched patients with traumatic cardiac arrest according to epinephrine administration (n = 634).

Characteristics	Epinephrine (n = 317)	No epinephrine (n = 317)	Standardized difference
Age, median (IQR), y	67 (47–77)	65 (43–77)	
≦17	3 (0.9%)	5 (1.6%)	−0.07
18–64	147 (46%)	152 (48%)	−0.03
≧65	167 (53%)	160 (51%)	0.04
Gender			0.01
Male	230 (73%)	232 (73%)	
Female	87 (27%)	85 (27%)	
Witness			−0.01
Unwitnessed	87 (27%)	89 (28%)	
Witnessed	230 (73%)	228 (72%)	
Bystander CPR			0.02
Any CPR	61 (19%)	64 (20%)	
No CPR	256 (81%)	253 (80%)	
**First rhythm**			
Ventricular fibrillation	2 (0.6%)	4 (1.3%)	−0.08
Pulseless ventricular tachycardia	1 (0.3%)	2 (0.6%)	−0.06
Pulseless electrical activity	127 (40%)	123 (39%)	0.03
Asystole	177 (56%)	180 (57%)	−0.02
Other	10 (3.2%)	8 (2.5%)	0.04
**Life support emergency medical personnel**			
Use of advanced life support devices	185 (58%)	188 (59%)	−0.02
Insertion of intravenous line	302 (95%)	302 (95%)	0
Time from call to arrival at scene	8 (6–10)	8 (6–11)	−0.08
Time from call to arrival at hospital	35 (28–44)	34 (27–44)	

IQR; interquartile range, CPR; cardiopulmonary resuscitation.

The subgroup and sensitivity analyses corroborated the results of the main analyses (Supplemental Tables 1 and 2, Figs 2 and 3).

## Discussion

### Brief summary

This study demonstrated that prehospital epinephrine administration in patients with TCA caused by traffic collisions was not associated with 1-month survival, whereas it was associated with prehospital ROSC.

### Comparison with previous studies

The results of the present study are consistent with those of a previous study, demonstrating that prehospital epinephrine administration in patients with TCA is associated with temporary ROSC^11^. Adrenergic system in the regulation of the cardiovascular system was well studied^22–24^. In patients with endogenous cardiac arrest, epinephrine administration has been reported to potentially increase the chance of ROSC through the arterioles mediated by α-adrenergic receptors^25^. This effect augmented temporal coronary blood flow and increased prehospital ROSC in patients with TCA^14,26^. However, the main cause of TCA was hemorrhagic shock^27^. A previous observational study reported that use of a vasopressor was associated with mortality in patients with traumatic hemorrhagic shock^28^. This study showed that the effect of epinephrine was limited and did not reach 1-month survival without definitive hemostatic treatment and/or blood transfusion management for hemorrhagic shock.

Previous article was that half of the patients who survived were penetrating trauma patients and this constituted a major bias^11^. The present study focused on blunt trauma patients with TCA caused by traffic collisions. In addition, our study analyzed data from a nationwide registry. Therefore, the sample size and generalizability of the findings are major strengths of this study. Besides, there were no biases related to hemostatic treatment because Japanese EMS systems could not do definitive hemostatic treatment.

In this study we used PS matching to control the variables including the time from the call to arrival at the scene of the accident and the time from the call to arrival at the hospital. Regarding clinical study in resuscitation area, the time dependent propensity score is thought to be more robust analysis^29,30^ and we included the time from the call to arrival at scene as one covarite for investigating the true effect of prehospital epinephrine administration for TCA.

### Possible explanations and implications

This study assessed the circumscription of prehospital management for patients with TCA in Japan. The mortality of patients with TCA varied depending on the previous reports^2–9^. The 1-month survival reported in the present study cohort was approximately 1%. This rate was lower compared with that reported in a recent systematic review^3^. Treatment options for EMS personnel in other countries extend to insertion of a chest tube, insertion of an intravenous line, and use of ALS devices^31^. Regarding fluid management, the usefulness of prehospital administration of red blood cells and blood plasma has been reported. Moreover, aggressive and varying prehospital management has been related to survival^32–35^. A previous report demonstrated that ALS performed by a treating physician was associated with survival among patients with TCA^10^. Therefore, it is recommended to use aggressive therapeutic options at the prehospital setting to achieve more favorable outcomes in patients with TCA.

The authors acknowledge the following limitations of this study. Firstly, as expected, survival from TCA was rare. First, the cause of mortality was not registered in the All-Japan Utstein Registry, therefore, we could not discuss what is the most severe injured organ and what in-hospital treatment was performed. In addition, there may be some medical caused cardiac arrest patients. These were strong bias of this study. Second, the rate of 1-month survival was approximately 1.0%. Considering that the goal of CPR is a favorable neurological outcome, we were unable to set the neurological outcome as an outcome of this study.

## Conclusion

Prehospital epinephrine administration in patients with TCA caused by traffic collisions was not associated with 1-month survival, but was beneficial in regards to prehospital ROSC. Currently, options for the prehospital treatment of trauma are limited.

## Supplementary information

Supplemental Table
