# Supplementary material for: Association of Prehospital Epinephrine Administration With Survival Among Patients With Traumatic Cardiac Arrest Caused By Traffic Collisions
Source: Sci Rep. 2019 Jul 9;9:9922. doi: 10.1038/s41598-019-46460-w (PMC6616542; doi:10.1038/s41598-019-46460-w)
Supplement: Supplementary file 1 — Supplemental Table [file 41598_2019_46460_MOESM1_ESM.docx]

Association of Prehospital Epinephrine Administration With Survival Among Patients With Traumatic Cardiac Arrest Caused By Traffic Collisions

Makoto Aoki, MD, PhD^1)^, Toshikazu Abe, MD, MPH^2,3)^, Kiyohiro Oshima, MD, PhD^1)^.

^1^Department of Emergency Medicine, Gunma University Graduate School of Medicine, Gunma, Japan.

^2^Department of General Medicine, Juntendo University, Tokyo, Japan.

^3^Department of Health Services Research, Faculty of Medicine, University of Tsukuba, Ibaraki, Japan.

Corresponding author:

Makoto Aoki, MD, PhD

Department of Emergency Medicine

Gunma University Graduate School of Medicine

3-39-22 Showa-town, Maebashi-city, Gunma

371-8541, Japan

Tel/Fax: +81-27-220-8541

E-mail: aokimakoto@gunma-u.ac.jp

Supplemental Table 1. Baseline characteristics of the propensity score-matched patients with traumatic cardiac arrest and with witness according to epinephrine administration (n=358)

| Characteristics | Epinephrine  (n=179) | No epinephrine  (n=179) | Standardized  difference |
| --- | --- | --- | --- |
| Age, median (IQR), y  ≦17  18-64  ≧65 | 68 (48-78)  0 (0%)  80 (45%)  99 (55%) | 66 (42-76)  1 (0.6%)  81 (45%)  97 (54%) | 0.00  -0.01  0.02 |
| Gender  Male  Female | 130 (73%)  49 (27%) | 133 (74%)  46 (26%) | 0.04 |
| Bystander CPR  Any CPR  No CPR | 28 (16%)  151 (84%) | 26 (15%)  153 (86%) | -0.03 |
| First rhythm  Ventricular fibrillation  Pulseless ventricular tachycardia  Pulseless electrical activity  Asystole  Other | 0 (0%)  0 (0%)  80 (45%)  96 (54%)  3 (1.7%) | 1 (0.6%)  0 (0%)  80 (45%)  94 (53%)  4 (2.2%) | 0.00  0.00  0.00  0.02  -0.04 |
| Life support emergency medical personnel  Use of advanced life support devices  Insertion of intravenous line  Time from call to arrival at scene  Time from call to arrival at hospital | 101 (56%)  168 (94%)  8 (6-10)  33 (27-41) | 99 (55%)  168 (94%)  8 (6-9)  33 (27-41) | 0.02  0.00  0.02 |

IQR; interquartile range, CPR; cardiopulmonary resuscitation

Supplemental Table 2. Baseline characteristics of the propensity score-matched patients with traumatic cardiac arrest including return of spontaneous circulation within 10 minutes according to epinephrine administration (n=358)

| Characteristics | Epinephrine  (n=333) | No epinephrine  (n=333) | Standardized  difference |
| --- | --- | --- | --- |
| Age, median (IQR), y  ≦17  18-64  ≧65 | 65 (40-76)  5 (1.5%)  161 (48%)  167 (50%) | 65 (42-77)  3 (0.9%)  158 (47%)  172 (52%) | 0.05  0.02  -0.03 |
| Gender  Male  Female | 261 (78%)  72 (22%) | 249 (75%)  84 (25%) | -0.09 |
| Witness  Unwitnessed  Witnessed | 96 (29%)  237 (71%) | 91 (27%)  242 (73%) | 0.03 |
| Bystander CPR  Any CPR  No CPR | 65 (20%)  268 (81%) | 66 (20%)  267 (80%) | 0.01 |
| First rhythm  Ventricular fibrillation  Pulseless ventricular tachycardia  Pulseless electrical activity  Asystole  Other | 7 (2.1%)  2 (0.6%)  142 (43%)  174 (52%)  8 (2.4%) | 4 (1.2%)  2 (0.6%)  135 (41%)  185 (56%)  7 (2.1%) | 0.06  0.00  0.04  -0.07  0.02 |
| Life support emergency medical personnel  Use of advanced life support devices  Insertion of intravenous line  Time from call to arrival at scene  Time from call to arrival at hospital | 179 (54%)  318 (96%)  8 (7-11)  37 (30-47) | 189 (57%)  318 (96%)  8 (6-11)  34 (28-46) | -0.06  0.00  0.08 |

IQR; interquartile range, CPR; cardiopulmonary resuscitation
